# Effect of individualized narrative nursing mode on recovery of elderly patients with fracture complicated with cerebrovascular accident

**DOI:** 10.1097/MD.0000000000036901

**Published:** 2024-01-19

**Authors:** Yurong Wang, Fang Zhang, Cuihua Li

**Affiliations:** a Department of Emergency, Tangshan Second Hospital, Tangshan, Hebei Province, China; b Department of Spine, The Second Hospital of Tangshan, Tangshan, Hebei Province, China; c Department of Nursing, Tangshan Second Hospital, Tangshan, Hebei Province, China.

**Keywords:** acute cerebrovascular accident, cognitive function, limb function, personalized narrative nursing, senile fracture

## Abstract

Fractures often occur in elderly patients. Osteoporosis caused by massive loss of calcium ions in the bones of elderly patients can easily lead to femoral fractures after suffering a low- and medium-energy injury. With the gradual entry of the aging society in China, the incidence of senile fracture is also gradually increasing. However, there is no report on the application of personalized narrative nursing to the mental health, cognitive function, and limb function recovery of elderly patients with fracture complicated with cerebrovascular accident, in order to enhance the cognitive level of elderly patients with fracture complicated with cerebrovascular accident. This study was specially conducted with a positive attitude toward the disease and improving the life quality. During July 2018 to July 2021, 80 elderly patients with fracture complicated with cerebrovascular accident cured were selected in our hospital. The patients were arbitrarily classified into an assigned control group (n = 40) and a study group (n = 40). The former received routine nursing, and the latter received personalized narrative nursing mode. The nursing satisfaction, functional independence scale (FIM), self-rating anxiety scale (SAS), self-rating depression scale (SDS), cognitive function, fracture healing time, length of hospital stays, and hospitalization expenses were compared. The study group had a satisfaction rate of 100.00%, while the control group had 87.50%. The nursing satisfaction of the study group was higher (*P* < .05). After 3 months of nursing, the FIM scores augmented. The FIM scores of upper and lower limbs in the study group were remarkably higher (*P* < .05). A decrease in SAS and SDS scores was observed. The SAS and SDS scores of the study group were lower (*P* < .05). Three months after discharge, the cognitive function score augmented. At 3 months after discharge, the study group had a higher cognitive function score (*P* < .05). The fracture healing time, length of stay, and cost of hospitalization in the study group were lower (*P* < .05). Personalized narrative nursing model can successfully enhance the mental health and cognitive function of elderly patients with fracture complicated with cerebrovascular accident, enhance the recovery of limb function, promote patients’ nursing satisfaction, and alleviate the economic burden.

## 1. Introduction

Fractures often occur in elderly patients. Osteoporosis caused by massive loss of calcium ions in the bones of elderly patients can easily lead to femoral fractures after suffering a low-and-medium-energy injury. With the gradual entry of the aging society in China, the incidence of senile fracture is also gradually increasing.^[1]^ Older patients tend to take more risks with their hands than younger fracture patients. Studies have shown that age, sex, health status, and concomitant diseases and other self-factors have been identified as potential risk factors affecting the prognosis of patients.^[2]^ Seventy-five percent of elderly patients were suffering from cardiovascular diseases, respiratory illnesses, cognitive impairments, and osteoporosis before they had surgery,^[3]^ which remarkably augmented postoperative complications and mortality.^[4]^ Because patients need absolute bed rest after fracture, it not only reduces the life quality but also makes them prone to complications of cerebral infarction and deep vein thrombosis in the lower limbs. According to statistics, the operative mortality rate of elderly patients older than 70 years old is 14%, and that of elderly patients older than 90 years old is 29.7%.^[5]^ Another study reported that in the United States, the mortality rate of elderly patients with fractures 1 month after operation was 6% to 9%, the 3-month mortality rate was 13% to 19%, and the postoperative 1-year mortality rate was 26% to 33%.^[6]^ Therefore, the perioperative management of elderly patients with fracture is very difficult.

The “social-psychological-biological” medical model has gradually become mature and has been widely used in various industries, of which the most important and prominent performance is the application in medicine. The problem that cardiovascular disease can easily lead to negative emotions has been concerned by many researchers and has been confirmed in many studies. Many factors will directly or indirectly affect the negative emotional level of elderly patients with fracture complicated with cerebrovascular accidents.^[7]^ At present, the main methods of intervention in the negative emotion of senile fracture complicated with cerebrovascular accident are drug therapy, traditional Chinese medicine therapy, health education, and psychological nursing. The use of drugs, such as duloxetine and citalopram, in the studies of some scholars led to an improvement in patients’ mood but was detrimental to their cardiac function. Traditional Chinese medicine therapy is a unique intervention method of chronic heart failure emotion in China, which can improve bad mood.^[8]^ Health education can improve the negative mood of elderly patients with fracture complicated with cerebrovascular accident, alleviate the emotional problems caused by patients’ lack of knowledge,^[9]^ and make up for the lack of patients’ knowledge. It tends to solve the emotional problems caused by patients’ lack of knowledge, but it is not suitable to deal with deep psychological problems. Psychological intervention also includes “two-heart nursing” model, music therapy, and relaxation therapy. They have some alleviating effect on the negative emotion of elderly patients with fracture complicated with cerebrovascular accident, but the concept of human care is insufficient. Narrative nursing originated from narrative medicine, which adheres to the concept and method of humanistic nursing. Through the implementation of the nursing and treatment technology of “narrative,” the connotation of humanistic nursing is reflected and sublimated in clinical work. In this nursing model, nurses respond and give feedback to patients by listening to their disease stories and life troubles, so that patients can feel a full of respect, understanding, and empathy. The aim is to advocate the integration of humanistic care into clinical nursing and truly solve patients’ confusion and troubles so that our nursing work becomes warmer.^[10]^ The application of narrative nursing can not only reduce the negative emotion of patients but also improve their life quality.^[11]^ The individual differences in disease and psychology of elderly patients with fractures complicated by cerebrovascular accidents place a high demand on individualized care. However, there is no report on the application of personalized narrative nursing to the mental health, cognitive function, and limb function recovery of elderly patients with fracture complicated with cerebrovascular accident, in order to enhance the cognitive level of elderly patients with fracture complicated with cerebrovascular accident. This study was specially conducted with a positive attitude toward the disease and improving the life quality.

## 2. Patients and methods

### 2.1. General information

During July 2018 to July 2021, 80 elderly patients with fracture complicated with cerebrovascular accident cured were selected in our hospital. A random sample of patients was classified into a control and a study group. The former accepted routine nursing, and the latter accepted personalized narrative nursing mode. In the control group, the age ranged from 61 to 89 years, with an average of 75.54 ± 3.42 years old, including 22 men and 18 women. Fracture disease consisted of 18 peoples of hip fracture, 12 peoples of knee fracture, and 10 peoples of pelvic fracture. Acute cerebrovascular accident included 22 peoples of cerebral hemorrhage and 18 peoples of cerebral infarction. In the study group, the age was 60 to 90 years old, with an average of 75.32 ± 3.42 years old, including 21 men and 19 women. Fracture disease consisted of 16 peoples of hip fracture, 14 peoples of knee fracture, and 10 peoples of pelvic fracture. Acute cerebrovascular accident included 21 peoples of cerebral hemorrhage and 19 peoples of cerebral infarction. According to the general data, there exhibited no statistical significance. We obtained informed consent from all patients, and the study was approved by The Second Hospital of Tangshan City Ethics Committee (No.: TSEY-LL-L20220059). All methods were carried out in accordance with relevant guidelines and regulations.

Selection criteria: (1) Age ≥ 65 years; (2) Fracture patients caused by low-energy external force (such as accidental fall); (3) The skin of the fracture site was intact, and there was no obvious local infection and systemic infection before operation; (4) Patients with cardiocerebrovascular complications after operation, or patients with previous history but new lesions after operation; (5) No history of mental illness, strong medical compliance before and after operation, good cooperation with follow-up; (6) The patients whose physical condition was allowed to operate after the comprehensive evaluation of each department; (7) The patients and their families knew about it.

Exclusion criteria: (1) Pathological fracture; (2) Intertrochanteric fractures caused by high-energy violence (such as car accidents or falls); (3) Patients with brain trauma, thoracic and abdominal trauma, autoimmune diseases, malignant tumors, and other diseases; (4) Patients with shock or severe underlying diseases at admission; (5) Patients who could not complete the observation such as automatic discharge and transfer occurred in the process of collecting medical history samples; (6) Noncompliant patients, incomplete data, and unwilling patients who refuse to cooperate with doctors.

#### 2.1.1. Treatment methods.

The patients were cured in accordance with the different types of accidental diseases of fracture complicated with cerebral hemorrhage. Among them, patients with hip fracture were treated with hip arthroplasty, patients with knee fracture were treated with knee arthroplasty, and patients with pelvic fracture were treated with internal fixation. At the same time, they were given symptomatic support treatment, such as hemostasis, reducing intracranial pressure, relieving brain edema, nourishing nerve, and so on.

During the admission process, the control group received routine nursing interventions in the department as well as disease guidance manuals, assessed the admission situation, patiently conducted health education for patients, and explained to patients the matters needing attention in disease-related self-management. Nurses provided individualized nursing care. There was a strong focus on health education when it came to education. The effect was observed after continuous nursing for 3 months.

According to protective motivation theory, nursing interventions were conducted on the study group based on the controls. Specifically, the following measures were taken:

1. The first step (attention stage): Patients were paid attention to and close nurse-patient relationship was established to win patients’ trust. Nursing objective: to establish an effective communication model based on the mutual trust between nurses and patients. The basic information was obtained about the patient through conversation to accurately assess the patient’s general condition and level of negative emotions. Nursing method: In a quiet communication space where patients could relax in the protection of their sense of security, we used positive and effective communication skills to talk to patients and achieve “empathy.” The patient’s family cultural background, social support background and personal interests were captured to support the next step of intervention. During the interview, team members should patiently listen to the patient’s “story” and encourage them to show the softest part of their heart as much as possible. In order to understand the patient’s psychological pain and the deepest expectations, you could ask the patient about the recent negative symptoms.
2. The second step (understanding stage): externalizing and deconstructing the problem. Nursing goal: To explore the impact of “confusion” on the patient’s life. Approach to care: Faced with the patient’s first description of confusion, the problem was “externalized” through a detailed description of how the problem arose and developed in the patient’s life. After sorting out the context of the “story,” we carried out anthropomorphic naming with the patients, such as “despair,” “drag,” “pain,” “elephant leg,” “irritability,” “anxiety,” and so on so that the problem was independent of the patient itself. The patient were allowed, as a bystander, to see the inner confusion and its effects from a different perspective.
3. The third step (reflection stage): Rewriting the story, through the patient’s reflection and review of past life processes, stimulates the patient’s strong inner potential. On the one hand, we should encourage patients to look for life “stories” that were very similar to the current situation, but have been neglected, and imagined how they overcome troubles at that time. To magnify and strengthen the positive role contained in exceptional events so that patients could truly identify with their inner strength. Second, through the power of example, through the sharing of successful medical records and anxiety among patients, we could gradually increase the courage of patients to face diseases and difficulties and enhance their self-confidence. In this process, playing the role of positive force and positive identity was conducive to the positive guidance of patients’ attitude toward life and individual behavior.
4. The fourth step (response stage): reconstruct the story and open up a new field of vision in life. Nursing objectives: analyze and rewrite the exceptional events explored in the reflection stage of patients, and connect the positive role with the present and even the future life, so as to give patients the courage to face a new life and open up new horizons in life as much as possible. Nursing methods: through this process, increase the hope of the patients, make the patients more eager to overcome themselves and move toward a new life, so we “rewrite” the life story of the patients.
5. The fifth step: proper use of “external witnesses” and “treatment documents” techniques nursing objectives: in the whole process of narrative nursing practice, carefully observe, and search for objects related to patients that are of great significance, such as letters and videos, to produce therapeutic documents for patients, allowing patients to compile their own values and life goals. Under the premise of patients’ consent, people who are of great significance in their lives can be involved not only by using the “eyes” and “statements” of witnesses to stimulate their own strength but also with the participation of family members, friends, and nursing staff, look for new stories and give patients confidence and encouragement. Nursing methods: choose appropriate “external witnesses” and “treatment documents” in accordance with the wishes of the intervention objects. For example, “external witnesses” can choose the medical staff in charge of the treatment, the patients’ closest parents, lovers, and patients in the same ward. Different types of document materials can be used as treatment documents, such as WeChat chats where patients encourage each other, photos of patients’ swollen legs taken every day during treatment, videos or short films documenting the patient’s treatment, medals, or certificates that the patient has received, and laboratory sheets that continue to improve since seeing a doctor to complete narrative nursing. In this case, before Uncle Li was discharged from the hospital, the treatment documents were made by collecting the hospitalization data of the uncle 27 times in the past 7 years so that the uncle could see the changes. The banner of “most cooperative treatment” was also one of the treatment documents awarded to the uncle. Through the witness of the head nurse, the attending physician, the responsible nurse, and the family members, the changes of the uncle can be seen. Finally, the uncle is encouraged to continue to cooperate with the treatment after he is discharged from the hospital. The effect was observed after continuous nursing for 3 months.

### 2.2. Observation indicators

#### 2.2.1. Satisfaction.

The satisfaction was observed after 3 months of nursing. In consultation with literature and experts, we designed a follow-up satisfaction survey for patients,^[12]^ a total of 10 items, and recorded patients’ satisfaction with follow-up management mode, health education, medical and nursing service, and appointment registration process. It was classified into 4 dimensions: very pleased, pleased, general, and displeased. Satisfaction rate = (very pleased + satisfactory + general) number of cases/total number of patients × 100.00%.

#### 2.2.2. FIM score.

After 3 months of nursing, the functional independence was evaluated again. Using the functional independence rating scale (FIM),^[13]^ FIM has been proposed by the American Association of Rehabilitation Medicine and the Society of Physical Medicine and Rehabilitation. Worldwide, patients were evaluated on this scale based on their daily activities. With 18 items encompassing 5 dimensions, including self-care ability, sphincter control, transfer, action ability, communication, and social cognition, resulting in a total score of 18 to 126. Patients with higher scores are more functionally independent. The FIM scale had good intra- and intergroup reliability, as well as good internal consistency.

#### 2.2.3. Self-rating anxiety scale and self-rating depression scale.

In addition to assessing anxiety and depression before nursing, a 3-month follow-up was also conducted. Self-rating anxiety scale (SAS),^[14]^ compiled by Zung in 1971, was a common scale for evaluating anxiety according to the situation of the last week, which was mainly adopted to assess the curative effect, not for diagnosis, and was often used to evaluate patients with clinical anxiety. The Cronbach alpha coefficient of this table was 0.826. There were 20 questions in SAS, which were scored by Likert-4 points. The whole part of the rough score multiplied by 1.25 was the standard score. A higher anxiety score indicates a greater degree of anxiety. Scores >50 were used as criteria for judging anxiety, 50 to 59 as mild anxiety, 60 to 69 as moderate anxiety, and >70 as severe anxiety. Self-rating depression scale (SDS) was compiled by Zung in 1965.^[15]^ According to the situation of the last week, it evaluated the symptoms and severity of depression and was widely used in emotional assessment, scientific research, and investigation. The Cronbach alpha coefficient of this table was 0.850. SDS included 20 items, which were scored by Likert-4 points. The whole part of the rough score multiplied by 1.25 was the standard score, and the score >50 was used as the criterion for judging depression, 50 to 60 as mild depression, 61 to 70 as moderate depression, and >70 as severe depression.

#### 2.2.4. Cognitive function score.

The cognitive function was observed before nursing and 3 months after discharge. LOTCA^[16]^ was a set of standardized neuropsychological tests for the evaluation of cognitive function. It could be used to evaluate the cognitive impairment of patients with brain injury caused by various causes, such as brain trauma, cerebrovascular accident, central nervous system developmental disorder, and tumor. The scores of the 26 test items all ranged from 1 (lowest) to 4 (highest), with the exception of some test items: (1) the scores of the 3 item classification tests ranged from 1 (lowest) to 5 (highest); and (2) the scores of the 2 directional test items ranged from 1 (lowest) to 8 (highest).

#### 2.2.5. Nursing index.

The fracture healing time, length of stay, and hospitalization expenses of the 2 groups were calculated. Fracture healing standard included with reference to X-ray examination, if the former fracture line appeared blurred or disappeared, it would be judged as fracture healing.

### 2.3. Statistical analysis

The data were analyzed statistically using SPSS 26.0. Mean ± standard deviation was adopted to describe measurement data, and (t-test) was adopted to test for statistical significance. For example, n (%) represented counting data, χ^2^ test was adopted. It was statistically significant when *P* < .05 was used.

## 3. Results

### 3.1. Comparison of nursing satisfaction

**Figure 1:**
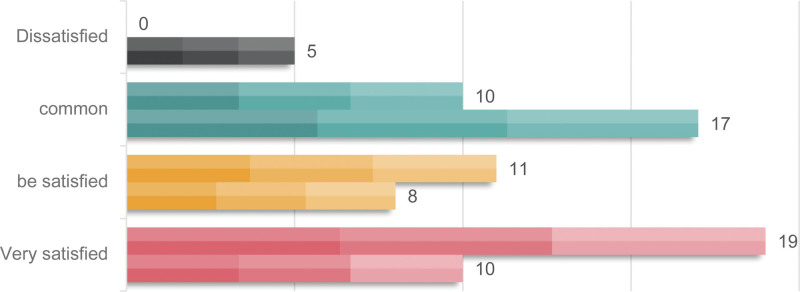
Comparison of nursing satisfaction.

With regard to nursing satisfaction, the study group was very pleased in 19 peoples, pleased in 11 peoples, general in 10 peoples, the satisfaction rate was 100.00%, while the control group was very pleased in 10 peoples, satisfactory in 8 peoples, general in 17 peoples, displeased in 5 peoples, the satisfaction rate was 87.50%. Study participants reported higher levels of nursing satisfaction (*P* < .05). All results were shown in Figure 1.

### 3.2. FIM score comparison

Prior to nursing, there exhibited no remarkable difference in FIM score (*P* > .05). After 3 months of nursing, the FIM scores of patients augmented, and the FIM scores of upper and lower limbs in the study group were remarkably higher (*P* < .05). All results were shown in Table 1.

**Table 1:** FIM scores [$\bar{x}\pm s$, points].

Group	N	Upper limb		Lower limb	
		Before nursing	After 3 mo of nursing	Before nursing	After 3 mo of nursing
C group	40	21.58 ± 3.22	48.59 ± 2.43*	9.54 ± 1.21	21.49 ± 3.43*
R group	40	21.54 ± 2.45	53.95 ± 3.32*	9.59 ± 1.54	25.34 ± 4.54*
*t*		0.062	8.239	0.161	4.279
*P*		>.05	<.01	>.05	<.01

* Indicated that this group is compared before and after nursing, *P* < .05.

FIM = functional independence scale.

### 3.3. Comparison of SAS and SDS scores

Prior to nursing, there exhibited no remarkable difference in the scores of SAS and SDS (*P* > .05). During the 3 months of nursing, SAS and SDS scores decreased. SAS and SDS scores were lower in the study group (*P* < .05). All results were shown in Table 2.

**Table 2:** SAS and SDS scores [$\bar{x}\pm s$, points].

Group	N	SAS		SDS	
		Before nursing	After 3 mo of nursing,	Before nursing	After 3 mo of nursing
C group	40	56.91 ± 2.32	51.29 ± 3.94*	59.64 ± 3.45	49.34 ± 4.76*
R group	40	56.29 ± 2.31	43.23 ± 2.32*	59.94 ± 3.56	41.49 ± 2.13*
*t*		1.197	11.148	0.382	9.520
*P*		>.05	<.01	>.05	<.01

* Indicated that this group is compared before and after nursing, *P* < .05.

SAS = self-rating anxiety scale, SDS = self-rating depression scale.

### 3.4. Comparison of cognitive function scores

Prior to nursing, there exhibited no remarkable difference in cognitive function score (*P* > .05). Three months after discharge, the cognitive function score augmented. At 3 months following discharge, the cognitive function score of the study group was higher (*P* < .05). All results were shown in Table 3.

**Table 3:** The cognitive function scores [$\bar{x}\pm s$, points].

Group	N	Before nursing	3 mo after discharge
C group	40	46.19 ± 2.32	60.19 ± 1.55*
R group	40	46.18 ± 2.54	74.29 ± 3.32*
*t*		0.018	24.338
*P*		>.05	<.01

* Indicated that this group is compared with that before nursing for 3 months, *P* < .05.

### 3.5. Comparison of nursing indexes

The fracture healing time, length of stay, and cost of hospitalization in the study group were lower (*P* < .05). All results were shown in Table 4.

**Table 4:** The nursing indicators of patients [$\bar{x}\pm s$].

Group	N	Fracture healing time (mo)	Length of stay (d)	Hospitalization expenses (10,000 yuan)
C group	40	4.45 ± 0.32	15.33 ± 2.33	1.95 ± 0.65
R group	40	3.38 ± 0.25	10.29 ± 2.32	0.54 ± 0.06
*t*		16.664	9.694	13.661
*P*		<.01	<.01	<.01

## 4. Discussion

As shown by China’s latest demographic statistics, there are 167 million people over 65, or 11.9% of the population.^[17]^ The elderly is often accompanied by varying degrees of osteoporosis and augmented bone fragility, which can lead to fractures with or without minor trauma. Similarly, the elderly also has a high incidence of cardiovascular and cerebrovascular diseases, which is not only a high-risk factor for fracture but also one of the most destructive complications of fracture. Fracture is easy to induce cardiocerebrovascular disease or aggravate the original cardiocerebrovascular disease. More and more evidence has shown that cardiocerebrovascular diseases, and fractures have common pathophysiological mechanisms and genetic factors. The elderly with previous cerebrovascular diseases is at high risk of fall fracture.^[18]^ According to the literature, about 73% of cerebrovascular disease survivors with mild to moderate disabilities experienced falls within the first 6 months after discharge, and about 1% to 15% of patients experienced fall-related fractures. Compared with the healthy control group, the risk of fracture in patients with cerebrovascular disease augmented 4 times, and the cumulative incidence of fracture in 2 years after the disease was as high as 5.7%. Hip fracture was the most obvious type of fracture, followed by wrist, rib, ankle and upper limb fractures.^[19,20]^ After fracture, it is easy to induce cardiocerebrovascular disease or aggravate the original cardiocerebrovascular disease, which not only brings a heavy burden to the family and society but also affects the life span and life quality of the elderly seriously. More and more evidence has shown that cardiocerebrovascular diseases and fractures have common pathophysiological mechanisms and genetic factors. After previous observation, we found that most of the elderly patients with fall fracture were complicated with cerebrovascular diseases. Compared with acute stroke and acute myocardial infarction, perioperative heart failure was more likely to be induced after fracture, the clinical manifestation was not typical. The treatment was more complicated. It is one of the serious perioperative complications, which can lead to prolonged hospitalization and augmented incidence of pneumonia, death, and other complications.^[21]^

Psychological and sociological systems widely employ narrative methods. According to Charan, narrative medicine, a combination of narratology and medicine, is a method of diagnosing and treating patients through narrative skills. Empathy and reflection are at the heart of this approach.^[22]^ Narrative therapy brings patients closer to expressing their feelings and thoughts through story telling. At the same time, patients should be helped visualize and specify the problems they are facing. Their objective perspective helps them construct a new story that alleviates the pain by focusing on it objectively.^[23]^ Narrative nursing is now more and more concerned with clinical nursing practice and carrying out by nursing professionals. “Disease narrative” can be carried out in a variety of ways through various intervention measures.^[24]^ Overseas, patient narratives have been implemented as a form of care in terminally ill, geriatric and cancer patients with good outcomes, and narrative care interventions have been carried out in adult cancer patients. Patients receiving narrative nursing interventions had a higher well-being index than those receiving control interventions. Using narrative nursing in mental health care, foreign scholars found that patients’ psychological well-being could be improved by alleviating mental tension and improving their medical condition.^[25]^ Domestic nursing scholars mostly have taken the significance of narrative nursing as the starting point, while Chinese scholars have found that fracture patients’ negative emotions can be improved by narrative nursing, and their confidence in fighting the disease can be enhanced.^[26]^ Chinese scholars have found that narrative nursing can successfully promote the life quality and psychological state of patients with cardiovascular disease, especially in emotional status, social/family status, functional status, and other dimensions. However, the improvement of physiological status is not obvious.^[27,28]^

Combined with the results, the nursing satisfaction of the study group was higher. Study group FIM scores for upper limbs and lower limbs were significantly higher than FIM scores for other limbs. Study participants scored lower on SAS and SDS. The cognitive function score of the study group was higher 3 months after discharge. The fracture healing time, length of stay, and cost of hospitalization in the study group were lower. Narrative nursing is to collect the basic materials of the patients to externalize, deconstruct and rewrite the patients’ conversations, and evaluate the narrative stories through the conversation with the patients. We also create narrative materials based on the patient’s mood and needs, and feedback on treatment interventions and outcome evaluations to discover the highlights of the patient’s illness experience.^[29,30]^ Provide treatment documentation based on the patient’s needs, thereby strengthening the patient’s positive self-perception, weakening the patient’s negative self-perception, and reinventing themselves.^[31]^ These bright spots are good things for patients, and helping patients to recall these beautiful things can not only relax the mood of patients. It also plays a role in training the patient’s memory, so that the patient’s brain can relax the mood while receiving stimulation, make the autonomic nerve excited, and improve the excitability of the hippocampus where the patient deals with learning and memory to improve the patient’s memory and executive ability.^[32]^ In addition, the study has shown that there is a negative correlation between psychological pain and neuropsychological performance in elderly patients with fracture complicated with cerebrovascular accident. Some studies have shown that depression remarkably affects the cognitive ability of many elderly patients with fracture complicated with cerebrovascular accident.^[33–35]^ Foreign scholars have found that depression often experienced by elderly patients with fracture complicated with cerebrovascular accident will affect cognitive ability, and insecurity will coexist with depression. A high level of insecurity will also affect the ability of cognitive adjustment.^[36]^ This study found that narrative nursing can improve patients’ depression, and patients’ sense of security can be improved by improving depression. Patients are more cooperative with the nurse and more willing to confide their true thoughts to the nurse, which can successfully enhance the accuracy of the intervention. At the same time, nurses use narrative nursing skills to change patients’ self-perceptions. This reduces the patient’s depression, reduces his or her insecurity and improves his or her cognitive ability.^[37]^ Then, narrative nursing can improve the patients’ body and mind from the aspects of psychology and cognition,^[38]^ and help the patients to maintain a good psychological state, which has a synergistic effect with good cognitive ability.^[39]^ Moreover, narrative nursing requires nurses to make use of the principle of “empathy,” stand in the patient’s point of view and put themselves in the patient’s shoes, and find the truest thoughts of the patients in their communication and interaction with the patients. Form an equal interpersonal relationship with patients, which helps to provide better nursing services for patients, and cultivate patients’ self-confidence by tapping their potential and existing internal strength. The patients’ self-cognition and improve patients’ cognitive ability will be reshaped.^[40–42]^ Eventually, patients should be guided to find that life not only has immediate difficulties but also unlimited possibilities in the future, change patients’ attitude toward life, and greet future life with more enthusiasm.^[43,44]^ The main limitation of this study is that the sample size of the study is small and the follow-up time is short. In future studies, we will expand the sample size and conduct a multi-center study to further explore better nursing programs.

## 5. Conclusion

To sum up, personalized narrative nursing model can successfully enhance the mental health and cognitive function of elderly patients with fracture complicated with cerebrovascular accident, promote the recovery of limb function, strengthen patients’ nursing satisfaction, and alleviate the economic burden.

## Author contributions

**Conceptualization:** Yurong Wang, Fang Zhang, Cuihua Li.

**Data curation:** Yurong Wang, Cuihua Li.

**Formal analysis:** Yurong Wang.

**Investigation:** Yurong Wang, Fang Zhang, Cuihua Li.

**Methodology:** Yurong Wang, Fang Zhang, Cuihua Li.

**Supervision:** Fang Zhang.

**Writing – original draft:** Yurong Wang, Fang Zhang, Cuihua Li.

**Writing – review & editing:** Yurong Wang, Fang Zhang, Cuihua Li.

## References

[1] Wang D Ge H Wang X. Complex fracture closure pressure analysis during shut-in: a numerical study. Energy Explor Exploit. 2022;40:1252–67.

[2] Grove W Goldin JA Breytenbach J. Separando la unión: de huellas digitales a esporas geno-digitales. Hum Geogr. 2022;15:609–12.

[3] Tropea A Tisano A Bruschetta A. Comparative FE biomechanical and microbial adhesion analyses on an implanted humerus. J Orthop. 2022;32:78–84. 35619601 10.1016/j.jor.2022.05.011 PMC9127272

[4] Lo CH Tang YHB. A case of subchondral insufficiency fracture of the knee at lateral femoral condyle treated with unicompartmental knee arthroplasty. Arthroplast Today. 2022;16:15–20. 35620586 10.1016/j.artd.2022.04.002 PMC9126744

[5] Rehm A Granger L Ngu A. Does compliance with British orthopaedic association standards for trauma and orthopaedics guidelines matter for displaced supracondylar fractures in children? The experience of a tertiary referral major trauma centre over a 35-year period. J Pediatr Orthop B. 2022;31:414–6. 35620840 10.1097/BPB.0000000000000959

[6] Sai BLNK Tambe P. Surface modified hollow glass microsphere reinforced 70/30 (wt/wt) PC/ABS blends: influence on rheological, mechanical, and thermo-mechanical properties. Compos Interfaces. 2022;29:617–41.

[7] Buxton GA. An irregular lattice spring model: uniform elasticity, grid refinement and isotropic crack propagation. Modelling Simul Mater Sci Eng. 2022;30:055002–408.

[8] Kithiia MW Munyasi MD Mutuli MS. Strength properties of surface modified Kenyan sisal fibres. J Nat Fibres. 2022;19:2277–87.

[9] Balcioğlu HE. Fracture behaviors of SiC particle filled and jute fiber reinforced natural composites. J Nat Fibres. 2022;19:2338–55.

[10] Bosco F Giustra F Faccenda C. Gorham-stout disease: a rare bone disorder. J Orthop Rep. 2022;1:100028–199.

[11] Mohamed S Pai SN. Mini fragment plating provides a middle ground between tension band wiring and conventional olecranon plating in the management of comminuted olecranon fractures: a case series. J Orthop Rep. 2022;1:100027–395.

[12] Choi YJ Watanabe N Takahashi K. Proposal of leaf chlorophyll content and its a/b ratio measurement method using a filter-free multiple wavelength sensor. Japan J Appl Phys. 2022;61:SD1041–53.

[13] Sampaio RFV Pragana JPM Bragança IMF. Revisiting the fracture forming limits of bulk forming under biaxial tension. Int J Damage Mech. 2022;31:198–200.

[14] Yang X Fan W Li Z. A continuum damage model for prediction of crack initiation life of pitting corrosion and fatigue. Int J Damage Mech. 2022;31:797–814.

[15] Miah MS Yu J Yang Y. Time-temperature-dependent mechanical durability analysis of short (glass) fiber-reinforced polyethylene terephthalate injection molding composites with weld line. Tex Res J. 2022;92:1923–39.

[16] Wakami T Fukunaga N Shimoji A. Surgical removal of a fractured guide wire for coronary angioplasty and coronary artery bypass grafting: report of a case. Kyobu Geka. 2022;75:931–5. 35618694

[17] Franekova L. Atypical femoral fracture after the withdrawal of bisphosphonates. J Clin Rheumatol. 2022;28:e675–6. 35616512 10.1097/RHU.0000000000001852

[18] Do HM Nguyen LH Dinh TT. Post-kyphoplasty secondary vertebral compression fractures in Vietnamese patients: a single-center prospective cross-sectional study. Ann Med Surg (Lond). 2022;78:38–45. 10.1016/j.amsu.2022.103756 PMC9127147 35620037

[19] Lai K Padilla BE Notrica DM. Nuss Procedure for pectus excavatum repair in a patient with osteogenesis imperfecta. J Pediatr Surg Case Rep. 2022;82:102311–411.

[20] Xu BH Liu K Zhao YH. Pullout resistance of densified wood dowel welded by rotation friction. J Mater Civ Eng. 2022;34:14–8.

[21] Boal MOK. “Small existential fractures and an interrogative relationship with the world”: an existentialist reading of Frances Hardinge’s a face like glass. Int Res Child Lit. 2022;15:19–24.

[22] Vaartjes TP Assink N Nijveldt RJ. Functional outcome after nonoperative management of tibial plateau fractures in skeletally mature patients: what sizes of gaps and stepoffs can be accepted? Clin Orthop Relat Res. 2022;480:2288–95. 35638902 10.1097/CORR.0000000000002266 PMC9653182

[23] Taha AY. Traumatic asphyxia in the young: report of two cases and literature review. Egypt J Forensic Sci. 2022;12:10–6.

[24] Li A Ding X Yu Z. Prediction model of fracture depth and water inrush risk zoning in deep mining coal seam floor. Environ Earth Sci. 2022;81:492–6.

[25] Kostiv RE Matveeva NY Kalinichenko SG. Localization of VEGF, TGF-β1, BMP-2, and apoptosis factors in hypertrophic nonunion of human tubular bones. Bull Exp Biol Med. 2022;173:160–8. 35624354 10.1007/s10517-022-05513-3

[26] Arcidiacono T Foligno N Vezzoli G. Efficacy of burosumab in healing a long-standing femoral fracture in an adult patient with X-linked hypophosphatemic rickets. Endocrine. 2022;77:566–7. 35633487 10.1007/s12020-022-03092-x

[27] Ogunleye TD Dugarte AJ Gilbertson JA. Reconstruction of complex acromion nonunions and fractures with a locking mesh plate. Tech Orthop. 2022;37:90–5.

[28] Hube LM Urbina BC Vargas GF. The modified posteromedial approach to posterior malleolar fractures. Tech Foot Ankle Surg. 2022;21:491–6.

[29] Rapp K Becker C Todd C. Association of two geriatric treatment systems on care home admission and mortality in patients with hip fracture. BMC Geriatr. 2022;22:191–5. 35624422 10.1186/s12877-022-03037-z PMC9145150

[30] Boiko DA Korabelnikova VA Gordeev EG. Integration of thermal imaging and neural networks for mechanical strength analysis and fracture prediction in 3D-printed plastic parts. Sci Rep. 2022;12:491–6. 35624225 10.1038/s41598-022-12503-y PMC9142534

[31] Karaytuğ K Alpan B Bayram S. Long-term results of different surgical options in the management of solitary enchondroma. ANZ J Surg. 2022;92:1809–13. 35621280 10.1111/ans.17796

[32] Dey Hazra RO Szewczyk K Ellwein A. Minimum 2-year results of the second-generation CFR-PEEK locking plate on the proximal humeral fracture. Eur J Orthop Surg Traumatol. 2022;33:1307–14. 35622161 10.1007/s00590-022-03298-9

[33] Sheng DL Burnham K Boutin RD. Ultrasound identifies first rib stress fractures: a case series in division I athletes. J Athl Train. 2022;58:664–8. 10.4085/1062-6050-0375.21 PMC10569245 35622951

[34] Zhao WG Zhang WL Zhang YZ. Characteristics of deep venous thrombosis in isolated lower extremity fractures and unsolved problems in guidelines: a review of recent literature. Orthop Surg. 2022;14:1558–68. 35633091 10.1111/os.13306 PMC9363729

[35] Hong JH Han MS Lee JK. Vertical split fracture of the vertebral body following oblique lumbar interbody fusion: a case report. Medicine (Baltimore). 2022;101:e29423–239. 35623075 10.1097/MD.0000000000029423 PMC9276454

[36] Chen M Guo T Zhang S. Numerical investigation into mechanical responses of frictional discontinuities with hydraulic fractures. Geomech Geophys Geo Energy Geo Res. 2022;8:193–8.

[37] Zhu C Zhu Z. Letter to the editor regarding, “Global incidence, prevalence, and disability of vertebral fractures: a systematic analysis of the global burden of disease study 2019” by Dong et al. Spine J. 2022;22:1070–185. 35598918 10.1016/j.spinee.2022.02.017

[38] Dong Y Li F. Reply to the letter to editor regarding, “Global incidence, prevalence, and disability of vertebral fractures: a systematic analysis of the global burden of disease study 2019”. Spine J. 2022;22:1071–186. 35598919 10.1016/j.spinee.2022.02.016

[39] Ahad A Haque A Armstrong A. The management of displaced humeral shaft fractures – a survey of UK shoulder and elbow surgeons. Shoulder Elbow. 2022;14:262–7. 10.1177/1758573220986940 PMC9121291 35599714

[40] Guevel B Gokaraju K Mohamed F. A comparative study of 6-week and 12-week Radiographic Union Scores for HUmeral fractures (RUSHU) as a predictor of humeral shaft non-union. Shoulder Elbow. 2022;14:294–302. 10.1177/17585732211033154 PMC9121295 35599708

[41] Thwaites S Thewlis D Hall K. Investigating and defining outcomes of suprapatellar versus infrapatellar intramedullary nailing of tibial shaft fractures: a protocol for a pilot randomised controlled trial. Pilot Feasibility Stud. 2022;8:303–6. 10.1186/s40814-022-01057-5 PMC9134682 35619162

[42] Kim CH Yoon YC Kang KT. The effect of cerclage wiring with intramedullary nail surgery in proximal femoral fracture: a systematic review and meta-analysis. Eur J Trauma Emerg Surg. 2022;48:4761–74. 35618854 10.1007/s00068-022-02003-z

[43] Kalir DM Shrira A Palgi Y. Feeling younger, rehabilitating better: reciprocal and mediating effects between subjective age and functional independence in osteoporotic fracture and stroke patients. Gerontology. 2022;69:109–17. 35613557 10.1159/000524885 PMC9808737

[44] Uzoigwe CE Riaz R Campbell-Jones F. Institutional use of spinal anaesthesia and hip fracture outcomes: analysis of the UK national hip fracture database. Br J Anaesth. 2022;128:e349–50. 35410789 10.1016/j.bja.2022.03.018

